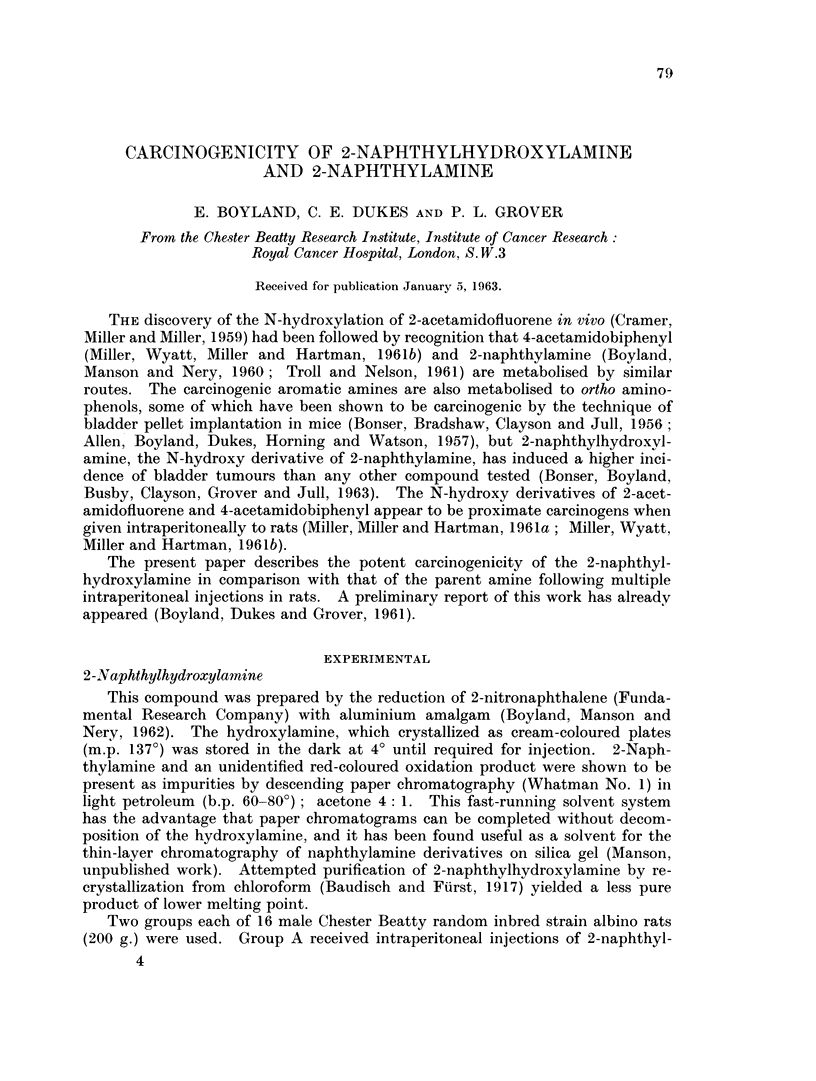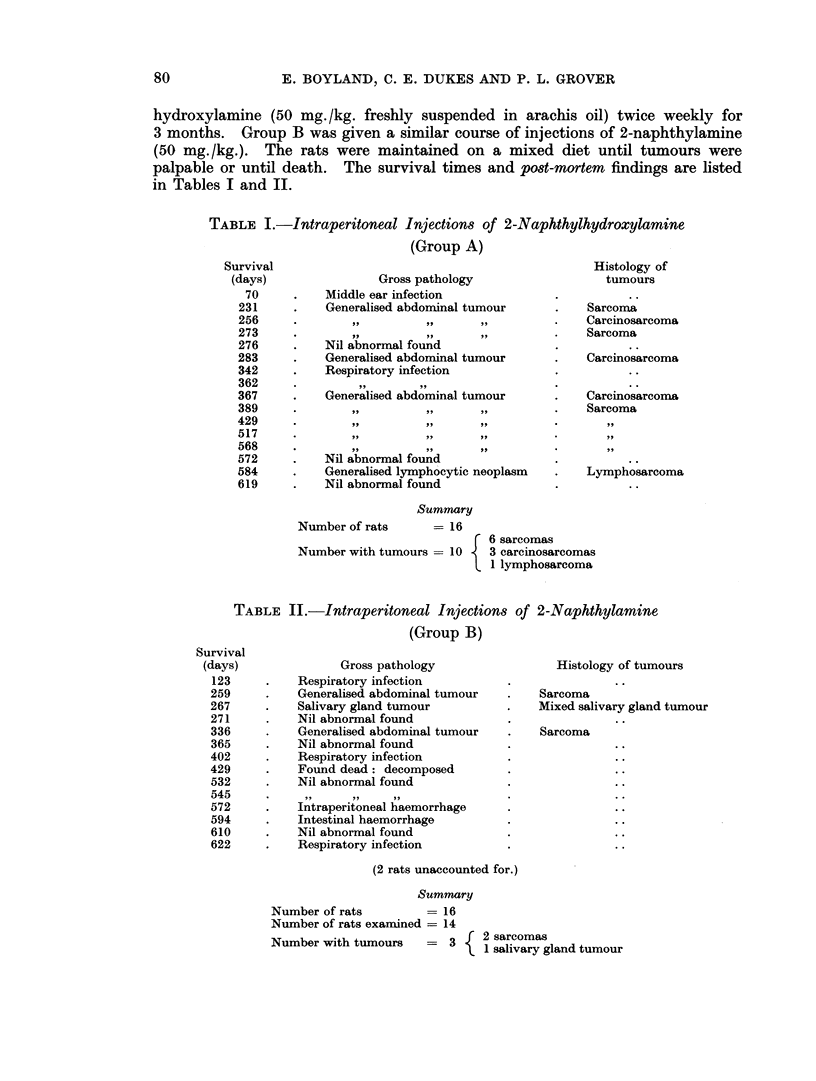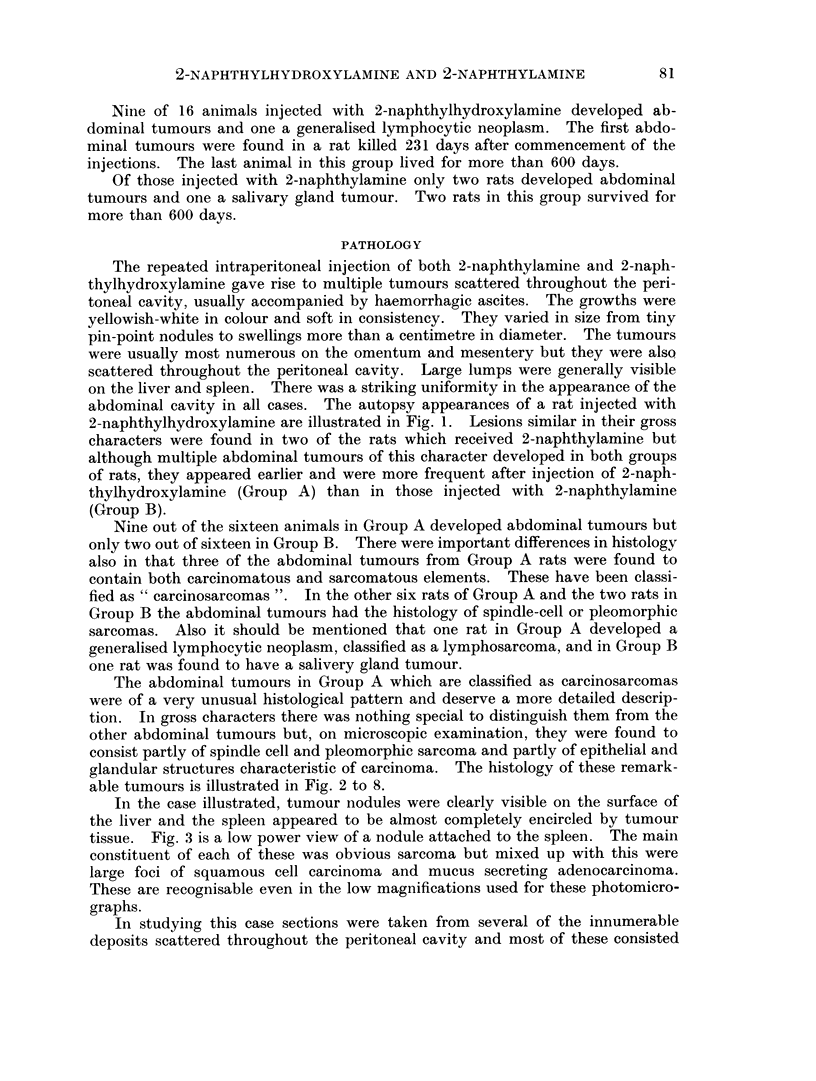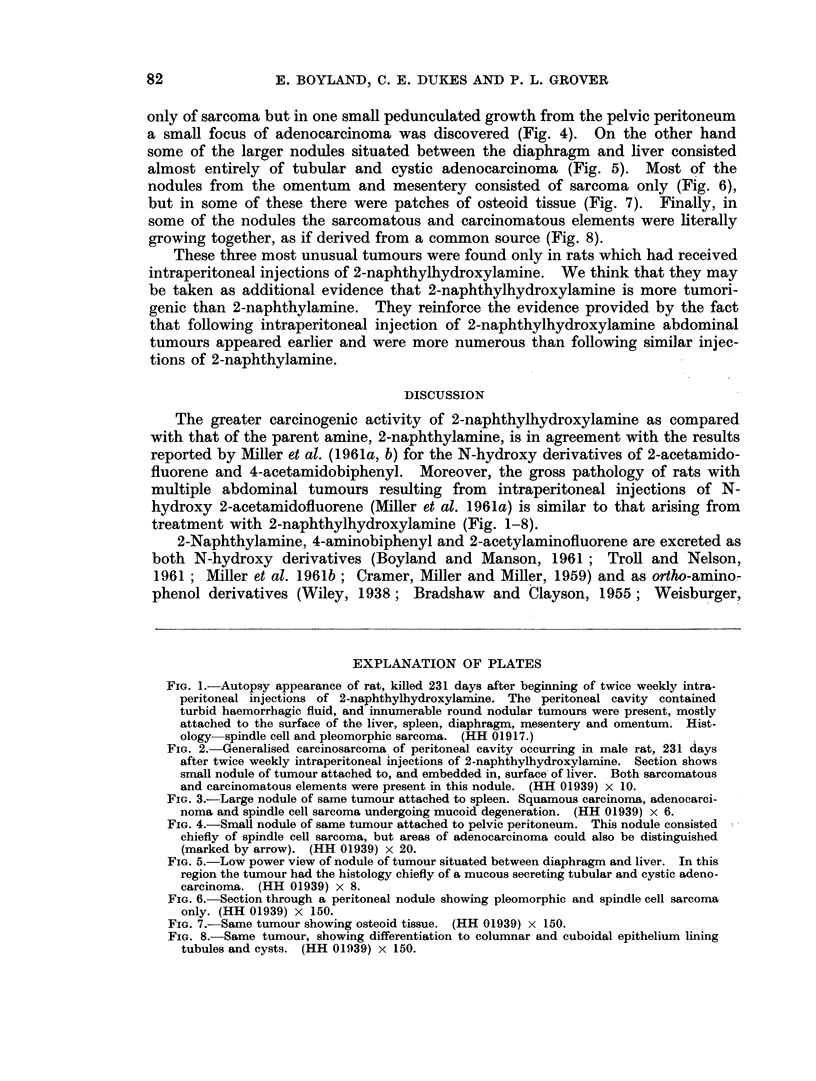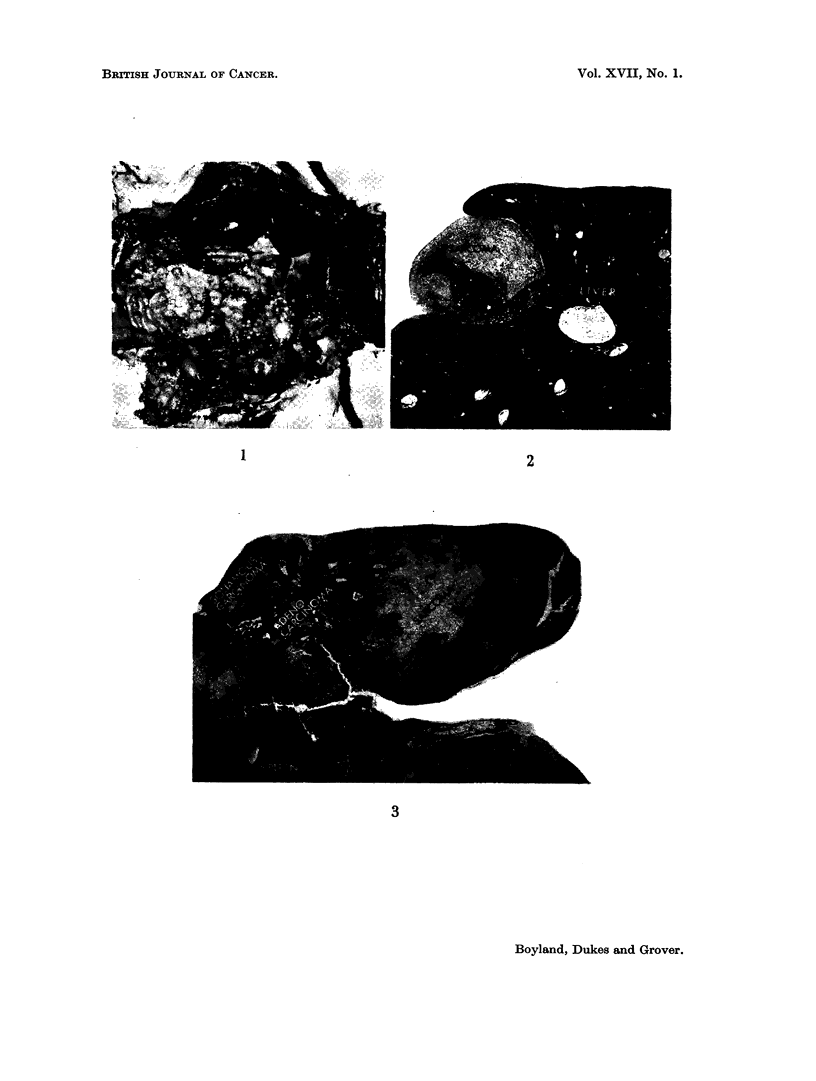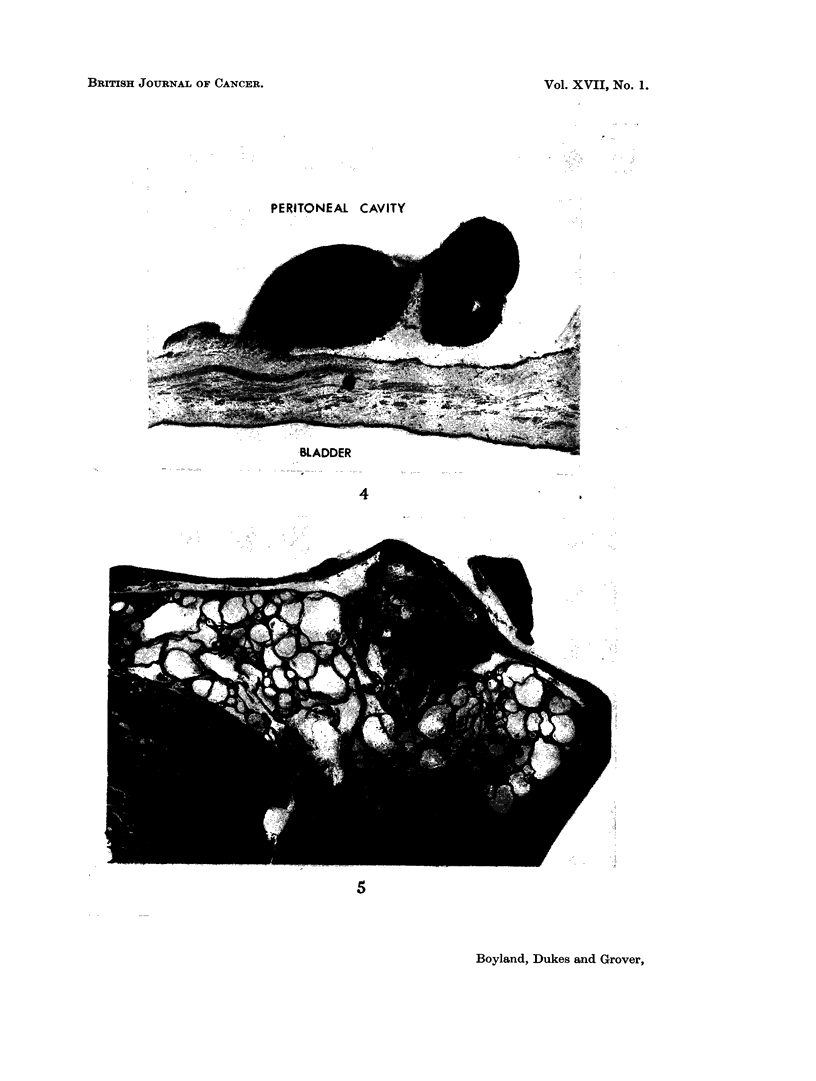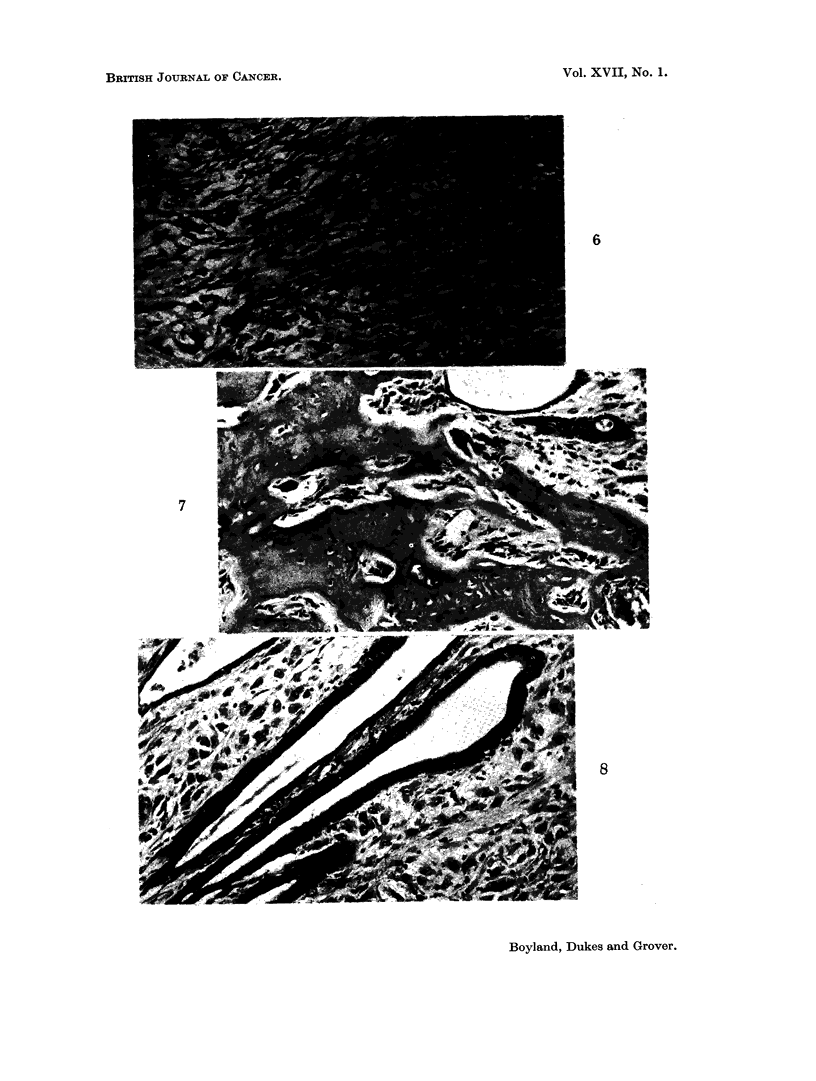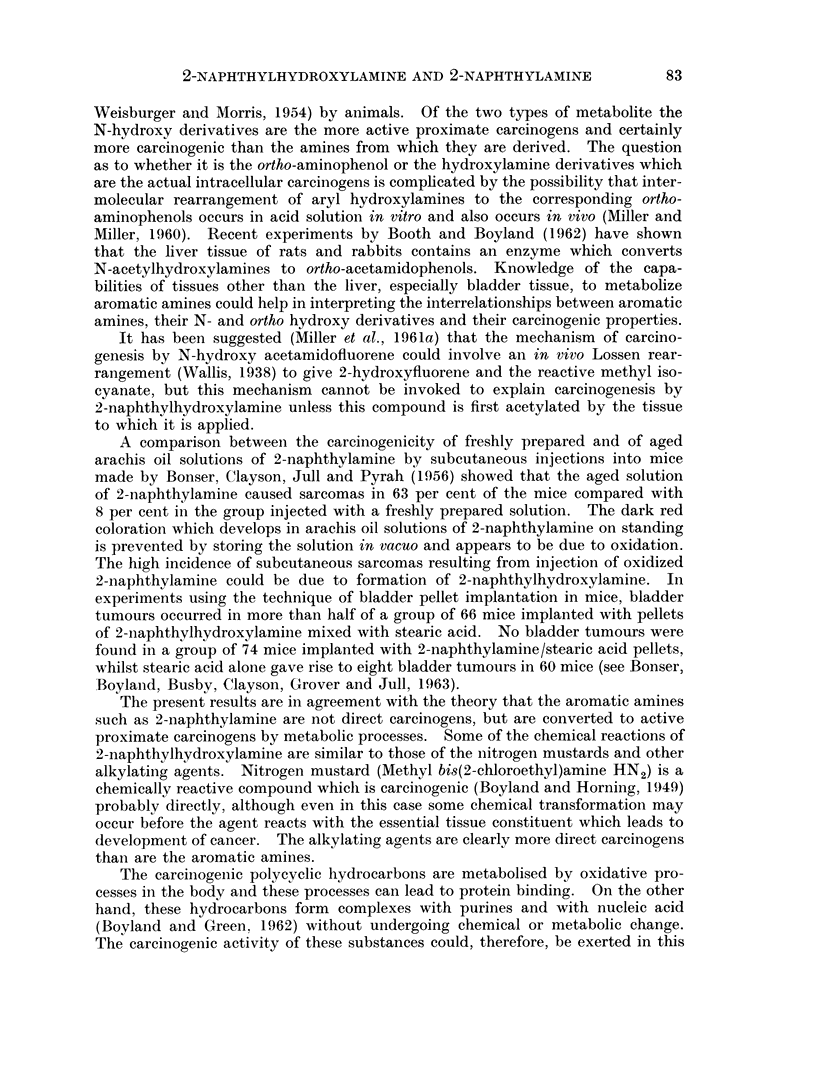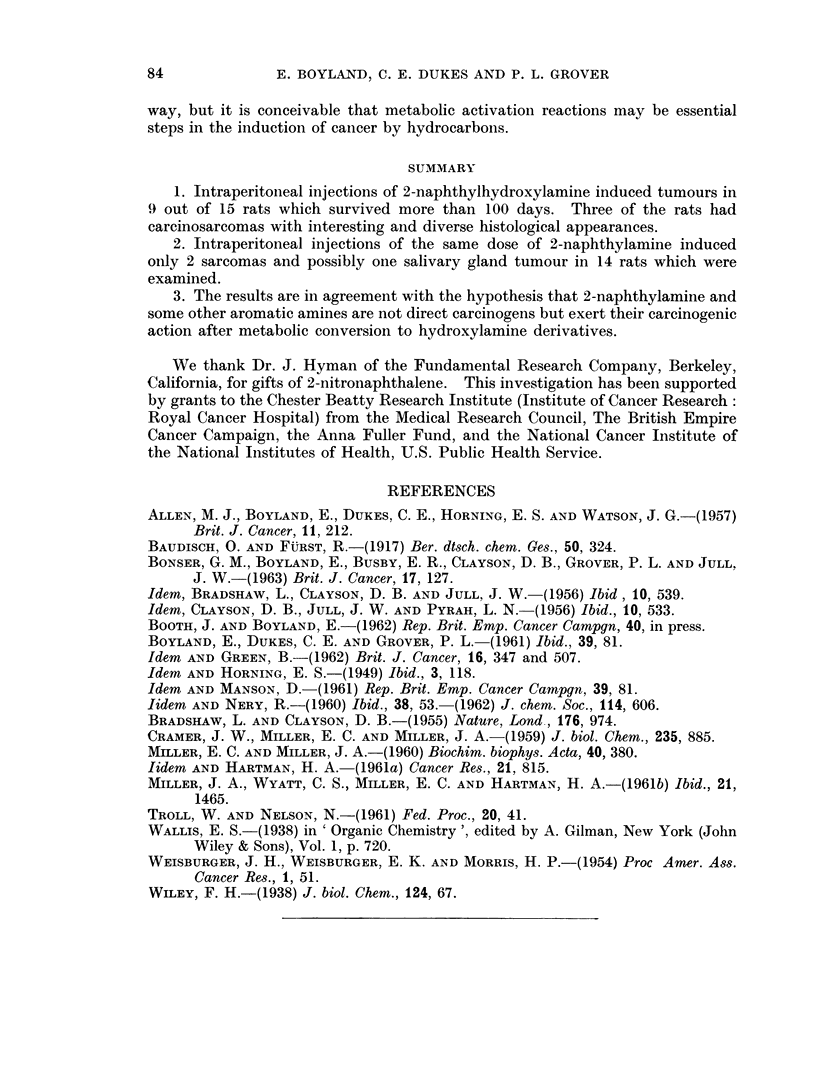# Carcinogenicity of 2-Naphthylhydroxylamine and 2-Naphthylamine

**DOI:** 10.1038/bjc.1963.12

**Published:** 1963-03

**Authors:** E. Boyland, C. E. Dukes, P. L. Grover

## Abstract

**Images:**


### CARCINOGENICITY OF 2-NAPHTHYLHYDROXYLAMINE AND 2-NAPHTHYLAMINE

E. BOYLAND, C. E. DUKES AND P. L. GROVER

*From the Chester Beatty Research Institute, Institute of Cancer Research : Royal Cancer Hospital, London, S.W.3*

Received for publication January 5, 1963.

THE discovery of the N-hydroxylation of 2-acetamidofluorene *in vivo* (Cramer, Miller and Miller, 1959) had been followed by recognition that 4-acetamidobiphenyl (Miller, Wyatt, Miller and Hartman, 1961*b*) and 2-naphthylamine (Boyland, Manson and Nery, 1960 ; Troll and Nelson, 1961) are metabolised by similar routes. The carcinogenic aromatic amines are also metabolised to *ortho* aminophenols, some of which have been shown to be carcinogenic by the technique of bladder pellet implantation in mice (Bonser, Bradshaw, Clayson and Jull, 1956 ; Allen, Boyland, Dukes, Horning and Watson, 1957), but 2-naphthylhydroxylamine, the N-hydroxy derivative of 2-naphthylamine, has induced a higher incidence of bladder tumours than any other compound tested (Bonser, Boyland, Busby, Clayson, Grover and Jull, 1963). The N-hydroxy derivatives of 2-acetamidofluorene and 4-acetamidobiphenyl appear to be proximate carcinogens when given intraperitoneally to rats (Miller, Miller and Hartman, 1961*a* ; Miller, Wyatt, Miller and Hartman, 1961*b*).

The present paper describes the potent carcinogenicity of the 2-naphthylhydroxylamine in comparison with that of the parent amine following multiple intraperitoneal injections in rats. A preliminary report of this work has already appeared (Boyland, Dukes and Grover, 1961).

### EXPERIMENTAL

### 2-*Naphthylhydroxylamine*

This compound was prepared by the reduction of 2-nitronaphthalene (Fundamental Research Company) with aluminium amalgam (Boyland, Manson and Nery, 1962). The hydroxylamine, which crystallized as cream-coloured plates (m.p. 137°) was stored in the dark at 4° until required for injection. 2-Naphthylamine and an unidentified red-coloured oxidation product were shown to be present as impurities by descending paper chromatography (Whatman No. 1) in light petroleum (b.p. 60–80°) ; acetone 4 : 1. This fast-running solvent system has the advantage that paper chromatograms can be completed without decomposition of the hydroxylamine, and it has been found useful as a solvent for the thin-layer chromatography of naphthylamine derivatives on silica gel (Manson, unpublished work). Attempted purification of 2-naphthylhydroxylamine by recrystallization from chloroform (Baudisch and Fürst, 1917) yielded a less pure product of lower melting point.

Two groups each of 16 male Chester Beatty random inbred strain albino rats (200 g.) were used. Group A received intraperitoneal injections of 2-naphthylhydroxylamine (50 mg./kg. freshly suspended in arachis oil) twice weekly for 3 months. Group B was given a similar course of injections of 2-naphthylamine (50 mg./kg.). The rats were maintained on a mixed diet until tumours were palpable or until death. The survival times and *post-mortem* findings are listed in Tables I and II.

TABLE I.—*Intraperitoneal Injections of 2-Naphthylhydroxylamine* (Group A)

| Survival (days) | Gross pathology | Histology of tumours |
|---|---|---|
| 70 | Middle ear infection | .. |
| 231 | Generalised abdominal tumour | Sarcoma |
| 256 | ,, ,, ,, | Carcinosarcoma |
| 273 | ,, ,, ,, | Sarcoma |
| 276 | Nil abnormal found | .. |
| 283 | Generalised abdominal tumour | Carcinosarcoma |
| 342 | Respiratory infection | .. |
| 362 | ,, ,, | .. |
| 367 | Generalised abdominal tumour | Carcinosarcoma |
| 389 | ,, ,, ,, | Sarcoma |
| 429 | ,, ,, ,, | ,, |
| 517 | ,, ,, ,, | ,, |
| 568 | ,, ,, ,, | ,, |
| 572 | Nil abnormal found | .. |
| 584 | Generalised lymphocytic neoplasm | Lymphosarcoma |
| 619 | Nil abnormal found | .. |

*Summary*

Number of rats = 16

Number with tumours = 10 { 6 sarcomas, 3 carcinosarcomas, 1 lymphosarcoma

TABLE II.—*Intraperitoneal Injections of 2-Naphthylamine* (Group B)

| Survival (days) | Gross pathology | Histology of tumours |
|---|---|---|
| 123 | Respiratory infection | .. |
| 259 | Generalised abdominal tumour | Sarcoma |
| 267 | Salivary gland tumour | Mixed salivary gland tumour |
| 271 | Nil abnormal found | .. |
| 336 | Generalised abdominal tumour | Sarcoma |
| 365 | Nil abnormal found | .. |
| 402 | Respiratory infection | .. |
| 429 | Found dead : decomposed | .. |
| 532 | Nil abnormal found | .. |
| 545 | ,, ,, ,, | .. |
| 572 | Intraperitoneal haemorrhage | .. |
| 594 | Intestinal haemorrhage | .. |
| 610 | Nil abnormal found | .. |
| 622 | Respiratory infection | .. |

(2 rats unaccounted for.)

*Summary*

Number of rats = 16

Number of rats examined = 14

Number with tumours = 3 { 2 sarcomas, 1 salivary gland tumour

Nine of 16 animals injected with 2-naphthylhydroxylamine developed abdominal tumours and one a generalised lymphocytic neoplasm. The first abdominal tumours were found in a rat killed 231 days after commencement of the injections. The last animal in this group lived for more than 600 days.

Of those injected with 2-naphthylamine only two rats developed abdominal tumours and one a salivary gland tumour. Two rats in this group survived for more than 600 days.

### PATHOLOGY

The repeated intraperitoneal injection of both 2-naphthylamine and 2-naphthylhydroxylamine gave rise to multiple tumours scattered throughout the peritoneal cavity, usually accompanied by haemorrhagic ascites. The growths were yellowish-white in colour and soft in consistency. They varied in size from tiny pin-point nodules to swellings more than a centimetre in diameter. The tumours were usually most numerous on the omentum and mesentery but they were also scattered throughout the peritoneal cavity. Large lumps were generally visible on the liver and spleen. There was a striking uniformity in the appearance of the abdominal cavity in all cases. The autopsy appearances of a rat injected with 2-naphthylhydroxylamine are illustrated in Fig. 1. Lesions similar in their gross characters were found in two of the rats which received 2-naphthylamine but although multiple abdominal tumours of this character developed in both groups of rats, they appeared earlier and were more frequent after injection of 2-naphthylhydroxylamine (Group A) than in those injected with 2-naphthylamine (Group B).

Nine out of the sixteen animals in Group A developed abdominal tumours but only two out of sixteen in Group B. There were important differences in histology also in that three of the abdominal tumours from Group A rats were found to contain both carcinomatous and sarcomatous elements. These have been classified as " carcinosarcomas ". In the other six rats of Group A and the two rats in Group B the abdominal tumours had the histology of spindle-cell or pleomorphic sarcomas. Also it should be mentioned that one rat in Group A developed a generalised lymphocytic neoplasm, classified as a lymphosarcoma, and in Group B one rat was found to have a salivery gland tumour.

The abdominal tumours in Group A which are classified as carcinosarcomas were of a very unusual histological pattern and deserve a more detailed description. In gross characters there was nothing special to distinguish them from the other abdominal tumours but, on microscopic examination, they were found to consist partly of spindle cell and pleomorphic sarcoma and partly of epithelial and glandular structures characteristic of carcinoma. The histology of these remarkable tumours is illustrated in Fig. 2 to 8.

In the case illustrated, tumour nodules were clearly visible on the surface of the liver and the spleen appeared to be almost completely encircled by tumour tissue. Fig. 3 is a low power view of a nodule attached to the spleen. The main constituent of each of these was obvious sarcoma but mixed up with this were large foci of squamous cell carcinoma and mucus secreting adenocarcinoma. These are recognisable even in the low magnifications used for these photomicrographs.

In studying this case sections were taken from several of the innumerable deposits scattered throughout the peritoneal cavity and most of these consisted only of sarcoma but in one small pedunculated growth from the pelvic peritoneum a small focus of adenocarcinoma was discovered (Fig. 4). On the other hand some of the larger nodules situated between the diaphragm and liver consisted almost entirely of tubular and cystic adenocarcinoma (Fig. 5). Most of the nodules from the omentum and mesentery consisted of sarcoma only (Fig. 6), but in some of these there were patches of osteoid tissue (Fig. 7). Finally, in some of the nodules the sarcomatous and carcinomatous elements were literally growing together, as if derived from a common source (Fig. 8).

These three most unusual tumours were found only in rats which had received intraperitoneal injections of 2-naphthylhydroxylamine. We think that they may be taken as additional evidence that 2-naphthylhydroxylamine is more tumorigenic than 2-naphthylamine. They reinforce the evidence provided by the fact that following intraperitoneal injection of 2-naphthylhydroxylamine abdominal tumours appeared earlier and were more numerous than following similar injections of 2-naphthylamine.

### DISCUSSION

The greater carcinogenic activity of 2-naphthylhydroxylamine as compared with that of the parent amine, 2-naphthylamine, is in agreement with the results reported by Miller *et al.* (1961*a*, *b*) for the N-hydroxy derivatives of 2-acetamidofluorene and 4-acetamidobiphenyl. Moreover, the gross pathology of rats with multiple abdominal tumours resulting from intraperitoneal injections of N-hydroxy 2-acetamidofluorene (Miller *et al.* 1961*a*) is similar to that arising from treatment with 2-naphthylhydroxylamine (Fig. 1–8).

2-Naphthylamine, 4-aminobiphenyl and 2-acetylaminofluorene are excreted as both N-hydroxy derivatives (Boyland and Manson, 1961 ; Troll and Nelson, 1961 ; Miller *et al.* 1961*b* ; Cramer, Miller and Miller, 1959) and as *ortho*-aminophenol derivatives (Wiley, 1938 ; Bradshaw and Clayson, 1955 ; Weisburger,

---

### EXPLANATION OF PLATES

Fig. 1.—Autopsy appearance of rat, killed 231 days after beginning of twice weekly intraperitoneal injections of 2-naphthylhydroxylamine. The peritoneal cavity contained turbid haemorrhagic fluid, and innumerable round nodular tumours were present, mostly attached to the surface of the liver, spleen, diaphragm, mesentery and omentum. Histology—spindle cell and pleomorphic sarcoma. (HH 01917.)

Fig. 2.—Generalised carcinosarcoma of peritoneal cavity occurring in male rat, 231 days after twice weekly intraperitoneal injections of 2-naphthylhydroxylamine. Section shows small nodule of tumour attached to, and embedded in, surface of liver. Both sarcomatous and carcinomatous elements were present in this nodule. (HH 01939) × 10.

Fig. 3.—Large nodule of same tumour attached to spleen. Squamous carcinoma, adenocarcinoma and spindle cell sarcoma undergoing mucoid degeneration. (HH 01939) × 6.

Fig. 4.—Small nodule of same tumour attached to pelvic peritoneum. This nodule consisted chiefly of spindle cell sarcoma, but areas of adenocarcinoma could also be distinguished (marked by arrow). (HH 01939) × 20.

Fig. 5.—Low power view of nodule of tumour situated between diaphragm and liver. In this region the tumour had the histology chiefly of a mucous secreting tubular and cystic adenocarcinoma. (HH 01939) × 8.

Fig. 6.—Section through a peritoneal nodule showing pleomorphic and spindle cell sarcoma only. (HH 01939) × 150.

Fig. 7.—Same tumour showing osteoid tissue. (HH 01939) × 150.

Fig. 8.—Same tumour, showing differentiation to columnar and cuboidal epithelium lining tubules and cysts. (HH 01939) × 150.

1

SARCOMA

CARCINOMA

LIVER

2

SQUAMOUS CARCINOMA

ADENO CARCINOMA

SARCOMA

SPLEEN

3

4

5

6

7

8

Boyland, Dukes and Grover.

Weisburger and Morris, 1954) by animals. Of the two types of metabolite the N-hydroxy derivatives are the more active proximate carcinogens and certainly more carcinogenic than the amines from which they are derived. The question as to whether it is the *ortho*-aminophenol or the hydroxylamine derivatives which are the actual intracellular carcinogens is complicated by the possibility that intermolecular rearrangement of aryl hydroxylamines to the corresponding *ortho*-aminophenols occurs in acid solution *in vitro* and also occurs *in vivo* (Miller and Miller, 1960). Recent experiments by Booth and Boyland (1962) have shown that the liver tissue of rats and rabbits contains an enzyme which converts N-acetylhydroxylamines to *ortho*-acetamidophenols. Knowledge of the capabilities of tissues other than the liver, especially bladder tissue, to metabolize aromatic amines could help in interpreting the interrelationships between aromatic amines, their N- and *ortho* hydroxy derivatives and their carcinogenic properties.

It has been suggested (Miller *et al.*, 1961*a*) that the mechanism of carcinogenesis by N-hydroxy acetamidofluorene could involve an *in vivo* Lossen rearrangement (Wallis, 1938) to give 2-hydroxyfluorene and the reactive methyl isocyanate, but this mechanism cannot be invoked to explain carcinogenesis by 2-naphthylhydroxylamine unless this compound is first acetylated by the tissue to which it is applied.

A comparison between the carcinogenicity of freshly prepared and of aged arachis oil solutions of 2-naphthylamine by subcutaneous injections into mice made by Bonser, Clayson, Jull and Pyrah (1956) showed that the aged solution of 2-naphthylamine caused sarcomas in 63 per cent of the mice compared with 8 per cent in the group injected with a freshly prepared solution. The dark red coloration which develops in arachis oil solutions of 2-naphthylamine on standing is prevented by storing the solution *in vacuo* and appears to be due to oxidation. The high incidence of subcutaneous sarcomas resulting from injection of oxidized 2-naphthylamine could be due to formation of 2-naphthylhydroxylamine. In experiments using the technique of bladder pellet implantation in mice, bladder tumours occurred in more than half of a group of 66 mice implanted with pellets of 2-naphthylhydroxylamine mixed with stearic acid. No bladder tumours were found in a group of 74 mice implanted with 2-naphthylamine/stearic acid pellets, whilst stearic acid alone gave rise to eight bladder tumours in 60 mice (see Bonser, Boyland, Busby, Clayson, Grover and Jull, 1963).

The present results are in agreement with the theory that the aromatic amines such as 2-naphthylamine are not direct carcinogens, but are converted to active proximate carcinogens by metabolic processes. Some of the chemical reactions of 2-naphthylhydroxylamine are similar to those of the nitrogen mustards and other alkylating agents. Nitrogen mustard (Methyl *bis*(2-chloroethyl)amine $HN_2$) is a chemically reactive compound which is carcinogenic (Boyland and Horning, 1949) probably directly, although even in this case some chemical transformation may occur before the agent reacts with the essential tissue constituent which leads to development of cancer. The alkylating agents are clearly more direct carcinogens than are the aromatic amines.

The carcinogenic polycyclic hydrocarbons are metabolised by oxidative processes in the body and these processes can lead to protein binding. On the other hand, these hydrocarbons form complexes with purines and with nucleic acid (Boyland and Green, 1962) without undergoing chemical or metabolic change. The carcinogenic activity of these substances could, therefore, be exerted in this way, but it is conceivable that metabolic activation reactions may be essential steps in the induction of cancer by hydrocarbons.

SUMMARY

1. Intraperitoneal injections of 2-naphthylhydroxylamine induced tumours in 9 out of 15 rats which survived more than 100 days. Three of the rats had carcinosarcomas with interesting and diverse histological appearances.

2. Intraperitoneal injections of the same dose of 2-naphthylamine induced only 2 sarcomas and possibly one salivary gland tumour in 14 rats which were examined.

3. The results are in agreement with the hypothesis that 2-naphthylamine and some other aromatic amines are not direct carcinogens but exert their carcinogenic action after metabolic conversion to hydroxylamine derivatives.

We thank Dr. J. Hyman of the Fundamental Research Company, Berkeley, California, for gifts of 2-nitronaphthalene. This investigation has been supported by grants to the Chester Beatty Research Institute (Institute of Cancer Research : Royal Cancer Hospital) from the Medical Research Council, The British Empire Cancer Campaign, the Anna Fuller Fund, and the National Cancer Institute of the National Institutes of Health, U.S. Public Health Service.

### REFERENCES

ALLEN, M. J., BOYLAND, E., DUKES, C. E., HORNING, E. S. AND WATSON, J. G.—(1957) *Brit. J. Cancer*, **11**, 212.

BAUDISCH, O. AND FÜRST, R.—(1917) *Ber. dtsch. chem. Ges.*, **50**, 324.

BONSER, G. M., BOYLAND, E., BUSBY, E. R., CLAYSON, D. B., GROVER, P. L. AND JULL, J. W.—(1963) *Brit. J. Cancer*, **17**, 127.

*Idem*, BRADSHAW, L., CLAYSON, D. B. AND JULL, J. W.—(1956) *Ibid* , **10**, 539.

*Idem*, CLAYSON, D. B., JULL, J. W. AND PYRAH, L. N.—(1956) *Ibid.*, **10**, 533.

BOOTH, J. AND BOYLAND, E.—(1962) *Rep. Brit. Emp. Cancer Campgn*, **40**, in press.

BOYLAND, E., DUKES, C. E. AND GROVER, P. L.—(1961) *Ibid.*, **39**, 81.

*Idem* AND GREEN, B.—(1962) *Brit. J. Cancer*, **16**, 347 and 507.

*Idem* AND HORNING, E. S.—(1949) *Ibid.*, **3**, 118.

*Idem* AND MANSON, D.—(1961) *Rep. Brit. Emp. Cancer Campgn*, **39**, 81.

*Iidem* AND NERY, R.—(1960) *Ibid.*, **38**, 53.—(1962) *J. chem. Soc.*, **114**, 606.

BRADSHAW, L. AND CLAYSON, D. B.—(1955) *Nature, Lond.*, **176**, 974.

CRAMER, J. W., MILLER, E. C. AND MILLER, J. A.—(1959) *J. biol. Chem.*, **235**, 885.

MILLER, E. C. AND MILLER, J. A.—(1960) *Biochim. biophys. Acta*, **40**, 380.

*Iidem* AND HARTMAN, H. A.—(1961*a*) *Cancer Res.*, **21**, 815.

MILLER, J. A., WYATT, C. S., MILLER, E. C. AND HARTMAN, H. A.—(1961*b*) *Ibid.*, **21**, 1465.

TROLL, W. AND NELSON, N.—(1961) *Fed. Proc.*, **20**, 41.

WALLIS, E. S.—(1938) in ' Organic Chemistry ', edited by A. Gilman, New York (John Wiley & Sons), Vol. 1, p. 720.

WEISBURGER, J. H., WEISBURGER, E. K. AND MORRIS, H. P.—(1954) *Proc Amer. Ass. Cancer Res.*, **1**, 51.

WILEY, F. H.—(1938) *J. biol. Chem.*, **124**, 67.